# Efficacy of bleomycin and sirolimus in inhibiting CD31^+^ endothelial cell proliferation in noninvoluting congenital hemangiomas

**DOI:** 10.3389/fcell.2025.1629770

**Published:** 2025-08-14

**Authors:** Yanan Li, Chuan Wang, Yi Li, Xinglong Zhu, Ji Bao, Yi Ji

**Affiliations:** ^1^ Department of Pediatric Surgery, West China Hospital of Sichuan University, Chengdu, China; ^2^ Department of Respiratory and Critical Care Medicine, State Key Laboratory of Respiratory Health and Multimorbidity, Institute of Respiratory Health, Frontiers Science Center for Disease-Related Molecular Network, West China Hospital, Sichuan University, Chengdu, China; ^3^ Department of Pathology, Institute of Clinical Pathology, Key Laboratory of Transplant Engineering and Immunology, NHC, West China Hospital, Sichuan University, Chengdu, China; ^4^ Department of Pediatric Surgery, West China Hospital of Sichuan University, Med-X Center for Informatics, Sichuan University, Chengdu, China

**Keywords:** noninvoluting congenital hemangiomas, CD31-positive endothelial cells, combination therapy, subcutaneous xenograft model, sirolimus

## Abstract

**Objective:**

Congenital hemangiomas are rare vascular anomalies that manifest at birth. Noninvoluting congenital hemangiomas present significant clinical challenges due to their persistence and associated complications. The mechanisms underlying congenital hemangiomas remain poorly understood, and current treatments have shown limited efficacy. This study aims to explore potential therapeutic strategies through the establishment of a stable cell model derived from noninvoluting congenital hemangiomas.

**Methods:**

Primary cells were isolated from noninvoluting congenital hemangioma tissue obtained from five patients, and CD31-positive endothelial cells were cultured and characterized. A subcutaneous xenograft model was established in nude mice to investigate tumorigenicity and evaluate the effects of various drugs, including bleomycin and sirolimus.

**Results:**

CD31-positive noninvoluting congenital hemangioma endothelial cells were successfully cultured and formed spheroids *in vitro*, demonstrating distinct morphological and immunohistochemical characteristics. When injected into nude mice, CD31-positive noninvoluting congenital hemangioma endothelial cells developed into tumors, whereas primary noninvoluting congenital hemangioma cells did not. Drug testing revealed that bleomycin and sirolimus effectively inhibited CD31-positive noninvoluting congenital hemangioma endothelial cells proliferation, with combination therapy showing significant tumor regression *in vivo*.

**Conclusion:**

The development of a stable cell model for noninvoluting congenital hemangiomas provides a valuable platform for understanding their pathogenesis and evaluating therapeutic options. The combination of bleomycin and sirolimus demonstrates promise as a novel treatment strategy, potentially improving outcomes for patients with noninvoluting congenital hemangiomas. Further studies are needed to explore the molecular mechanisms involved and to assess the efficacy across different congenital hemangioma subtypes.

## 1 Introduction

Congenital hemangiomas (CHs) represent uncommon vascular anomalies in pediatric populations that arise *in utero* and present as fully formed lesions at birth. Unlike infantile hemangiomas (IHs), which exhibit well-defined proliferating and involuting stages, CHs are classified into three distinct subtypes: rapidly involuting congenital hemangiomas (RICHs), noninvoluting congenital hemangiomas (NICHs), and partially involuting congenital hemangiomas (PICHs) (Ayturk et al., 2016; Wei et al., 2023; Qiu et al., 2024a). RICH generally gradually regresses postnatally and typically resolves entirely within 6–14 months (Boon et al., 1996). In contrast, NICHs persist throughout a child’s development. PICH initially presents characteristics similar to those of RICH but ceases regressing at a certain point, resulting in residual lesions that are challenging to differentiate from NICH (Enjolras et al., 2001; Mulliken and Enjolras, 2004). Recent research has indicated that some NICHs are not completely static and may exhibit a secondary proliferative phase after several years (Hua et al., 2021).

CHs may result in a range of complications, such as permanent deformities, ulceration, hemorrhage, obstruction of vital organs, and congestive heart failure (Boon et al., 1996). The pathogenesis of CH remains poorly understood. Propranolol, a nonselective β-blocker and the current first-line treatment for IH, has demonstrated limited efficacy in the treatment of CH, as corroborated by our previous research (Wei et al., 2023; Vildy et al., 2015). For NICHs and PICHs, interventional embolization has shown limited effectiveness, frequently necessitating surgical excision of the lesions. Surgical intervention for NICH lesions located on the face or perineal region can result in considerable trauma to the patient. Furthermore, for extensive NICH lesions that may be associated with functional impairments, there is currently no safe and effective treatment available (Tripathi et al., 2021; Cohen-Cutler et al., 2021; Olsen et al., 2020).

There is an imperative need to elucidate the underlying mechanisms of CH to identify potential therapeutic targets. However, research on its etiology and drug validation is significantly limited by the absence of stable cell models. This study investigated a novel strategy for culturing primary cells from NICHs and successfully established a subcutaneous xenograft model of CH in nude mice by isolating CD31-positive endothelial cells (CD31+NICHECs) from NICHs through flow cytometry. Using both cell and animal models, we subsequently discovered that the combination of bleomycin and sirolimus may exert an inhibitory effect on NICH.

## 2 Materials and methods

### 2.1 Study design

Following approval from the Committee on Clinical Investigation, NICH samples from five patients (Table 1), were collected from the Pediatric Surgery Department at West China Hospital, Sichuan University. The diagnosis was confirmed by the Department of Pathology. Informed consent and publication consent were obtained from patients’ parents. The Ethics Committee of West China Hospital approved this study (Approval No: 2019(1085)). The IACUC and the Animal Experiment Center at Sichuan University also approved all the animal procedures (Approval No: 20220307040).

**TABLE 1:** The clinical characteristics of the patients.

Patient number	Age (m)	Sex	Size (cm)	Biopsy site	Successful isolation
1#	11	Female	1.5 × 2.5	Right thigh	Yes
2#	48	Female	3 × 3	Right thigh	No
3#	28	Female	2 × 3	Left shoulder	Yes
4#	16	Male	3 × 4	Back	No
5#	19	Male	2 × 2.5	Back	Yes

### 2.2 Cell isolation and culture

The samples were minced under sterile conditions and coated with vascular-specific Matrigel. They were cultured in endothelial basal medium (EBM-2, Lonza, Walkersville, MD) supplemented with 20% fetal bovine serum, penicillin, and streptomycin. After 14 days, the cells were digested with trypsin to obtain primary NICH cells. Primary NICH cells were subsequently labeled with CD31 antibodies (ab9498, 1:50) and sorted into CD31^+^ cells via flow cytometry. The isolated CD31^+^ NICHECs were cultured in EBM-2 medium supplemented with 1% penicillin‒streptomycin (HyClone) and 10% fetal bovine serum (Gibco, NY, United States). Cultures were maintained in a humidified environment with 5% CO_2_ at 37 °C.

### 2.3 Rapid cultivation of CD31^+^ NICHECs spheroids

Initially, specialized glass culture dishes (with a length‒width ratio of 1.36) were prepared by treating them with Sigmacoat (Sigma, MO, United States) and subjecting them to high-temperature sterilization to ensure thorough disinfection. Subsequently, 10 mL of culture medium was added to each dish, and 3 million CD31^+^ NICHECs were seeded per dish. To facilitate optimal cellular development and interaction, the dishes were agitated at a consistent rate of 10 cycles per minute. Concurrently, an EVOS™ XL Core microscope was used to monitor the morphology of the spheroids in detail. Next, the spheroids were fixed in 10% neutral buffered formalin for subsequent immunohistochemical analysis.

### 2.4 Subcutaneous tumor formation in nude mice

Primary CD31^+^ NICHECs were suspended in a matrix gel and subsequently injected subcutaneously into the right thigh region of 6-week-old male nude mice at a concentration of 1 × 107 cells per mouse. On the 20th day postinjection, the animals were euthanized, the subcutaneous graft tumors were harvested, and the tumors were fixed in 10% neutral buffered formalin for subsequent immunohistochemical analysis.

### 2.5 Immunohistochemistry

Using immunohistochemistry, we investigated the distinctive characteristics of CD31^+^ NICHECs, subcutaneous tumors, and NICH tumor tissues. Following fixation in 4% neutral formalin, the samples were sectioned and stained with hematoxylin and eosin. We selected diagnostic markers for NICH, including platelet endothelial cell adhesion molecule-1 (CD31, ab256569, ab9498 and ab76533, 1:200, Abcam), hematopoietic progenitor cell antigen (CD34, ab81289, 1:100, Abcam), and glucose transporter type 1 (GLUT-1, ab115730, 1:100, Abcam), for immunohistochemical analysis. Additionally, Ki-67 (ab16667, 1:100, Abcam) was utilized to assess cellular proliferation.

### 2.6 Evaluation of drug effects

An appropriate quantity of CD31^+^ NICHECs was seeded into a 96-well culture plate and incubated for 12 h. The cells were subsequently treated with various concentrations of axitinib, cabozantinib, propranolol, bleomycin, and sirolimus (10 nM, 50 nM, 100 nM, 200 nM, 500 nM, 1 μM, or 2 μM). After an incubation period of 48 h, CCK-8 working solution was added to assess the optical density (OD) of the cells.

### 2.7 Statistical analysis

Statistical analyses for this study were conducted via SPSS version 21.0 software (SPSS, Inc., Chicago, IL, United States). All the quantitative variables are presented as the means ± standard deviations. Dunnett’s test was utilized for pairwise quantitative comparisons, whereas analysis of variance (ANOVA) was employed for analyses involving multiple groups. A p value of less than 0.05 was considered to indicate statistical significance.

## 3 Results

### 3.1 Isolation and culture of NICH cells

**FIGURE 1:**
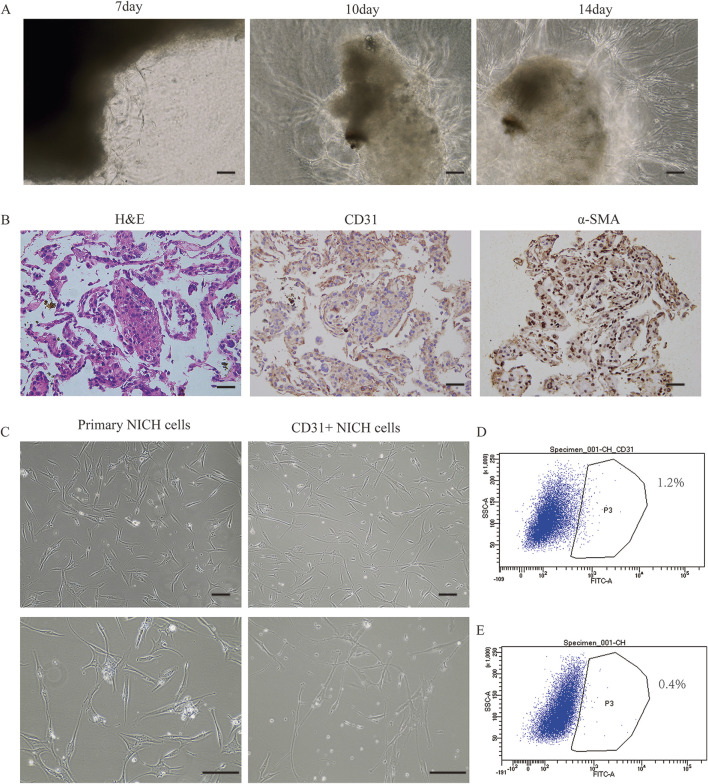
Isolation and cultivation of NICH cells. **(A)** Progressive derivation of cells from the NICH tumor mass at days 7, 10, and 14. **(B)** Hematoxylin and eosin (H&E), CD31, and α-smooth muscle actin (α-SMA) staining of cells progressively derived from the NICH tumor mass. **(C)** Primary NICH cells and CD31^+^ NICHECs. **(D)** CD31-positive cell sorting. **(E)** Negative control for cell sorting. Scale bars = 100 μm.

By employing a vascular-specific matrix gel to encapsulate NICH tissue, we successfully cultured primary NICH cells derived from the tumor mass (Figure 1A). Immunohistochemical staining revealed that a minor fraction of the primary NICH cells isolated from the tumor were CD31 positive, whereas the majority were α-SMA positive (Figure 1B). Morphologically, the CD31^+^ NICHECs appeared more elongated and larger than the unsorted NICH cells did (Figure 1C). Flow cytometry analysis corroborated these findings, revealing that only approximately 1.2% of the cells were CD31 positive (Figure 1D). Subsequently, three strains of CD31^+^ NICHECs were successfully isolated from these samples.

### 3.2 Identification of CD31^+^ NICHECs

**FIGURE 2:**
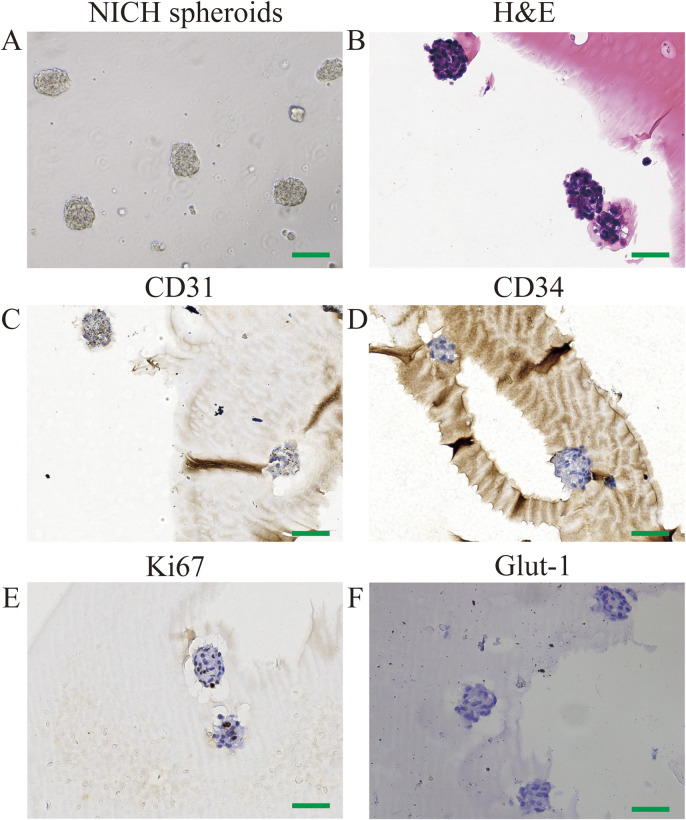
Identification of CD31^+^ NICHECs. **(A)** CD31^+^ NICHEC cell spheres; **(B)** H&E staining; **(C)** positive CD31 staining; **(D)** negative CD34 staining; **(E)** few Ki67-positive cells; **(F)** negative GLUT-1 staining. Scale bars = 100 μm.

Using a rotary cell culture system, CD31^+^ NICHECs were cultured into spheroid formations (Figure 2A). Postfixation, hematoxylin and eosin staining revealed densely packed cell clusters (Figure 2B). Immunohistochemical analysis revealed that the cells within the spheroids were positive for CD31 staining (Figure 2C), negative for CD34 (Figure 2D) and contained a limited number of Ki67-positive cells (Figure 2E). The cells within the spheroids were also negative for GLUT-1 staining (Figure 2F).

### 3.3 Subcutaneous tumor formation in nude mice

**FIGURE 3:**
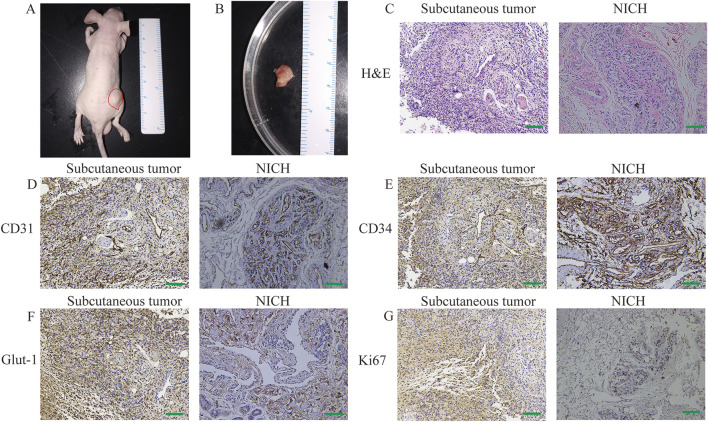
*In vivo* tumor formation. **(A)** Subcutaneous tumor formation in nude mice. **(B)** Tumor dissection, **(C)** H&E staining, **(D)** CD31 staining, **(E)** CD34 staining, **(F)** GLUT-1 staining, and **(G)** Ki67 staining. Scale bars = 100 μm.

The clonally expanded primary NICH cells and CD31^+^ NICHECs were resuspended in Matrigel and subsequently injected subcutaneously into nude mice. Ten days after the injection of CD31^+^ NICHECs, a subcutaneous tumor progressively developed in the right thighs of the mice (Figures 3A,B). HE staining revealed that the subcutaneous tumor tissue was highly similar to NICH tissue (Figure 3C). In contrast, the injection of primary NICH cells did not result in tumor formation. After 20 days, immunohistochemical analysis of the subcutaneous tumors in the nude mice revealed strong positive staining for CD31 (Figure 3D) and CD34 (Figure 3E), akin to NICH tissue. However, only a limited number of NICH cells were positive for GLUT-1 expression, whereas GLUT-1 was strongly positive in subcutaneous tumors. (Figure 3F). Additionally, a limited number of cells in NICH tissue were Ki67 positive, whereas xenograft tumors in nude mice demonstrated strong Ki67 positivity (Figure 3G).

### 3.4 Drug validation utilizing CD31^+^ NICHECs

**FIGURE 4:**
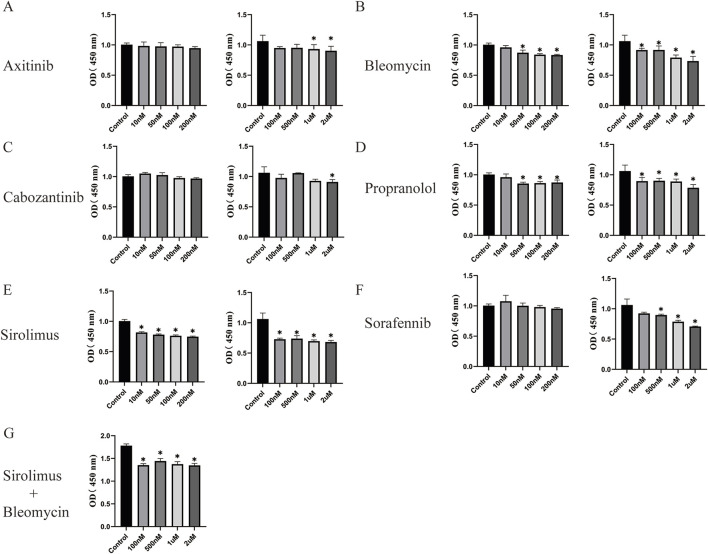
Use of drugs utilizing CD31^+^ NICHECs. **(A)** Axitinib, **(B)** bleomycin, **(C)** cabozantinib, **(D)** propranolol, **(E)** sirolimus, **(F)** sorafenib, **(G)** sirolimus plus bleomycin. *P < 0.05, compared with the control, five samples per group.

Our study demonstrated that axitinib (Figure 4A) inhibited the proliferation of CD31^+^ NICHECs only at concentrations as high as 2 μM. Bleomycin at a concentration of 50 nM had an inhibitory effect on CD31^+^ NICHECs proliferation, with a marginal increase in inhibition observed at higher concentrations (Figure 4B). Cabozantinib also innhibited the proliferation of CD31^+^ NICHECs only at concentrations as high as 2 μM (Figure 4C). In contrast, propranolol began to have inhibitory effects at 50 nM; however, further increases in concentration did not significantly augment this effect (Figure 4D). Conversely, sirolimus inhibited proliferation at a concentration of 10 nM, with a gradual increase in inhibitory efficacy as the concentration increased (Figure 4E). Sorafenib inhibited CD31^+^ NICHECs proliferation at a concentration of 500 nM (Figure 4F). When the combined effect of sirolimus and bleomycin was evaluated, a baseline concentration of 50 nM sirolimus was used, and the combination significantly suppressed CD31^+^ NICHECs proliferation. However, further increases in concentration did not result in a substantial increase in the inhibitory effect (Figure 4G).

### 3.5 *In vivo* investigations of pharmacological impacts on NICHs

**FIGURE 5:**
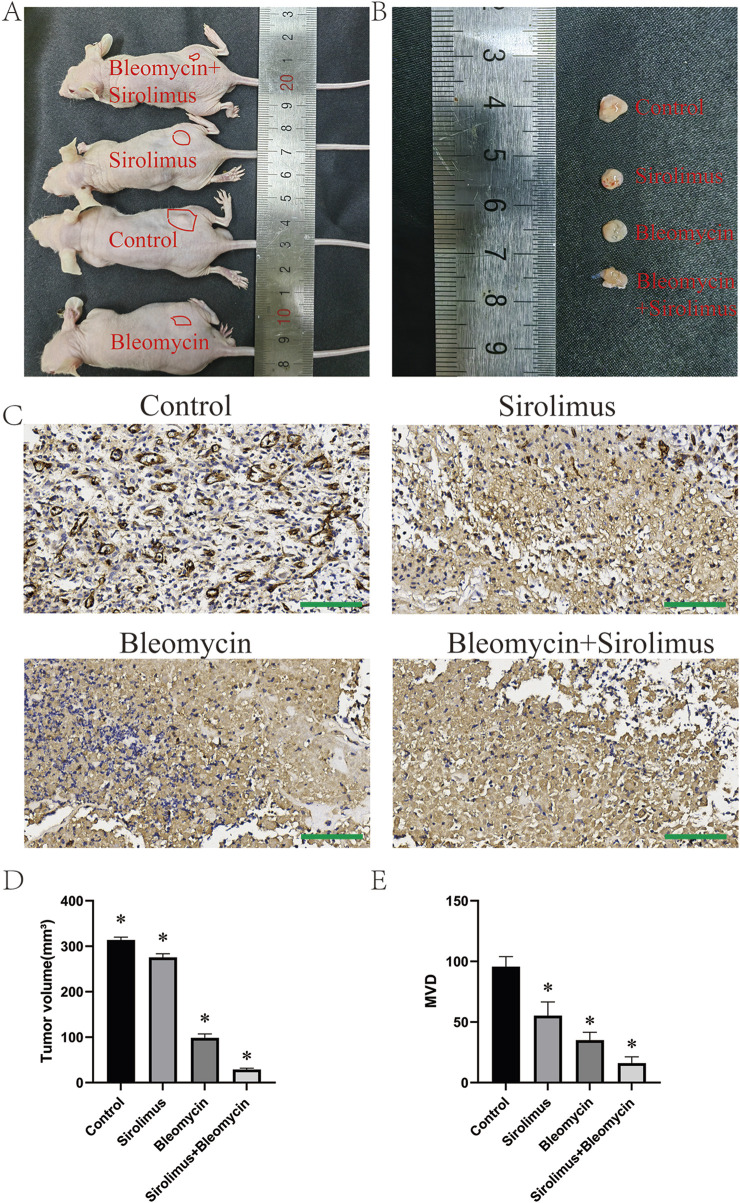
*In vivo* drug validation experiments. **(A)** Subcutaneous tumor formation in nude mice, **(B)** dissected tumor specimen, **(C)** CD31 staining, **(D)** tumor volume, **(E)** tumor vascular density. *P < 0.05, compared with the control, three samples per group. Scale bars = 100 μm.

In the subcutaneous tumor formation experiment conducted with nude mice, following a 20-day feeding period, bleomycin, sirolimus, or their combination were administered via injection into the subcutaneous tumors over the course of 1 week (Figure 5A). The mice were subsequently euthanized, and tumor specimens were harvested for volumetric analysis. The findings indicated that the administration of bleomycin, sirolimus, or their combination resulted in significant regression of the subcutaneous tumors (Figure 5B). Subsequent analysis of microvessel density (MVD) differences via immunohistochemical (IHC) staining with the CD31 antibody revealed that all treatment modalities resulted in a reduction in vascular density within the tumors (Figure 5C). Notably, combination therapy had the most pronounced effect (Figures 5D,E).

## 4 Discussion

The absence of cellular and animal models for NICH has significantly impeded research on its pathogenesis and the screening of potential therapeutics. However, the rapid advancement of three-dimensional (3D) *in vitro* cell culture technology offers a promising alternative. These 3D *in vitro* cell models can closely replicate the structure and function of *in vivo* tumor tissues, thereby partially reconstructing the tumor microenvironment *in vitro*. This advancement markedly enhances tumor cell viability, tissue morphology, genotypic stability, function, and drug metabolism (Hamilton and Rath, 2019). We are dedicated to developing and optimizing 3D cell models for vascular anomalies. In our prior research, we successfully developed a high-throughput microtumor model derived from IHs via micropattern arrays. This advancement offers a more stable and efficient experimental framework for investigating the mechanisms underlying IH and for conducting drug validation (Li et al., 2022). With the swift advancement of regenerative medicine, organoids have emerged as a significant research platform for drug development and have the potential to address the limitations inherent in traditional models (Miyoshi et al., 2020; O'Connell and Winter, 2020). Organoids are intricate three-dimensional constructs derived from stem cells or organ-specific progenitor cells through self-organization processes (Rossi et al., 2018). Owing to their composition of multiple cell types and the presence of multicellular organ-like structures, organoids closely mimic the architecture and functionality of *in vivo* organs, thereby providing a near-replica of the human internal environment (Wang et al., 2017). In recent years, numerous organoid models have been developed and validated as robust platforms for high-throughput drug validation and mechanistic investigations (Gabriel et al., 2021; Mun et al., 2019). In a prior study, through continuous optimization of the cell culture system, we successfully established NICH organoids (Wei et al., 2023). However, our attempts to transplant these NICH organoids into the subcutaneous tissue of nude mice did not result in the successful formation of xenograft tumors. In contrast, CD31-positive hemangioma endothelial cells (CD31^+^ HemECs), which are frequently utilized as cell models in IH research, have been successfully employed to construct subcutaneous xenograft tumor models in nude mice (Li et al., 2022; Ji et al., 2012; Yang et al., 2023). In this study, we employed a cell-specific matrix gel for the encapsulation of NICH tumors, facilitating the successful culture of primary NICH cells. Primary NICH cells did not demonstrate the capacity to induce tumor formation in immunodeficient nude mice. Using flow cytometry sorting, we isolated CD31^+^ NICHECs and subsequently established a subcutaneous xenograft tumor model of NICH in nude mice. In the initial cell suspension, the fraction of cells with genuine and robust tumorigenic potential, capable of forming tumors subcutaneously in nude mice, is likely diminished by a substantial presence of non-tumorigenic or weakly tumorigenic cells. The presence of non-tumorigenic cells, such as normal fibroblasts and immune cells, within the population may contribute to the creation of a microenvironment that inhibits tumor growth. Through the application of flow cytometry to isolate CD31^+^ cells from primary NICH cells, it was determined that CD31^+^ cells comprised merely about 1.2% of the population. This model offers a robust experimental platform for mechanistic investigations of NICHs.

The pathogenesis of CH, with potential etiological factors, including genetic dysregulation and mechanical injury during embryonic development, remains incompletely understood. Comparative studies have demonstrated that congenital hemangiomas exhibit distinct gene expression profiles and mutations relative to IHs, indicating that they represent separate biological entities (Picard et al., 2008). Notably, endothelial cells in IH exhibit high expression levels of GLUT-1, a marker typically absent in NICH (Konanur et al., 2022). GLUT1, a glucose transporter, mediates the translocation of glucose from the bloodstream into cells (Veys et al., 2020). The expression of GLUT1 is modulated by hypoxia-inducible factor-1 (HIF-1), a transcription factor that orchestrates angiogenesis through the activation of genes such as vascular endothelial growth factor (VEGF) and basic fibroblast growth factor (bFGF) (Li et al., 2021). By augmenting glucose uptake, GLUT1 facilitates angiogenesis, supplying essential energy and metabolites, including lactate and pyruvate, which serve as signaling molecules to stimulate endothelial cell proliferation and migration (Veys et al., 2020). In proliferating IHs, GLUT1 is robustly expressed, which diminishes during the involution phase (Rodriguez Bandera et al., 2021). In contrast to IHs, which proliferate postnatally, CHs undergo maturation *in utero*. Interestingly, a restricted number of cells within the NICH group displayed positive GLUT-1 expression, in contrast to the strong positivity observed for GLUT-1 in subcutaneous tumors. These findings suggest that elevated GLUT1 expression in the early stages of CH development may facilitate its development. Furthermore, insulin-like growth factor-2 (IGF-2) has been demonstrated to be markedly upregulated in IHs and is correlated with the expression levels of vascular endothelial growth factor receptor-2 (VEGFR-2) (Ritter et al., 2002). Compared with IHs, CHs consistently present elevated expression levels of vascular endothelial growth factor receptor 1 (VEGFR-1) and comparatively reduced expression of IGF-2. Several studies have identified mutations in the GNAQ and GNA11 genes in specific cases of CH. These genetic mutations are implicated in the aberrant activation of the MAPK/MEK/Ras signaling pathway, which is intimately associated with abnormal vascular proliferation and angiogenesis (Ayturk et al., 2016; Cohen-Cutler et al., 2021; Funk et al., 2016). Our previous transcriptomic sequencing data revealed significant enrichment of the PI3K/AKT, MAPK, and RAS signaling pathways in NICH, and subsequent drug validation experiments revealed that propranolol, sirolimus, and trametinib did not have substantial inhibitory effects on NICH organoids (Wei et al., 2023). Consequently, this study aimed to further investigate the effects of axitinib, cabozantinib, sorafenib, propranolol, bleomycin, and sirolimus on CD31^+^ NICHECs. Our study demonstrated that the combination of sirolimus and bleomycin significantly inhibited the proliferation of CD31^+^ NICHECs. Furthermore, intratumoral injection of bleomycin, sirolimus, or their combination in subcutaneous tumors in nude mice revealed that the combination therapy substantially suppressed tumor growth. These findings suggest that the combined use of sirolimus and bleomycin may represent a promising therapeutic strategy for NICH.

Bleomycin is an antineoplastic agent extensively utilized in the management of diverse cutaneous lesions, including infantile hemangiomas. Its mechanism of action involves binding to DNA, resulting in strand breaks and the subsequent inhibition of cellular proliferation. This mechanism is particularly important in the treatment of hemangiomas, as their growth is intimately linked to the proliferation of vascular endothelial cells (Vanhooteghem, 2022). Bleomycin induces apoptosis in endothelial cells, thereby diminishing vascular formation and expansion, which facilitates the reduction and regression of hemangiomas (Guo et al., 2024). Furthermore, bleomycin exerts immunomodulatory effects by augmenting the immune response against tumor cells, thereby contributing to the regression of hemangiomas (Lee et al., 2017). The local injection of bleomycin allows direct targeting of the affected area, which minimizes systemic side effects and increases the drug concentration at the lesion site, thus increasing therapeutic efficacy (Shao et al., 2016). Research indicates that the combination of bleomycin with other treatments, such as oral propranolol, can improve treatment outcomes and shorten the duration of therapy (Thayal et al., 2012). Sirolimus, an inhibitor of the mammalian target of rapamycin (mTOR), has been extensively investigated for its therapeutic potential across a range of diseases and has achieved notable prominence in the management of kaposiform hemangioendothelioma (KHE) in recent years. Its mechanism of action involves the inhibition of the mTORC1 signaling pathway, leading to a reduction in cellular proliferation and survival (Ji et al., 2022). This mechanism is particularly critical in the treatment of KHE, an aggressive vascular tumor frequently associated with severe complications such as the Kasabach–Merritt phenomenon (KMP). Furthermore, sirolimus has significant antiangiogenic effects, effectively suppressing the formation of new blood vessels (Qiu et al., 2024b). Sirolimus has demonstrated potential in enhancing patient outcomes through its immunomodulatory effects (Zhou et al., 2023). Specifically, it inhibits the activation and proliferation of T cells, thereby mitigating inflammatory responses. This mechanism can alleviate symptoms and improve the prognosis of patients diagnosed with KHE. Furthermore, in patients with KHE associated with KMP, sirolimus has been shown to be effective in increasing platelet counts and reducing the risk of severe hemorrhagic events (Ji et al., 2017). Additionally, sirolimus has antilymphangiogenic properties, which are crucial in the treatment of KHE, as this condition may involve aberrant lymphatic development. In recent years, researchers have investigated the potential of integrating sirolimus with other pharmacological agents to augment its therapeutic efficacy. These combination strategies not only have the capacity to enhance antitumor effects but also improve patient outcomes by modulating immune responses and optimizing the pharmacokinetic properties of drugs (Ji et al., 2022; Cohen et al., 2012; Gangadhar et al., 2011). In conclusion, the synergistic therapeutic mechanisms of sirolimus and bleomycin likely involve multiple biological pathways, including the inhibition of cellular proliferation, augmentation of DNA damage, enhancement of immune responses against tumor cells, and modifications within the tumor microenvironment.

This study is subject to several limitations. The subcutaneous tumor model in nude mice utilized cells derived from only three patients with NICH, which may not have sufficiently captured the heterogeneity and complexity of CH patients. The research has focused predominantly on NICHs, thereby excluding RICHs and PICHs. This narrow focus restricts a comprehensive understanding of the pathophysiological mechanisms and therapeutic responses across the various subtypes of CH. Our study demonstrated that both bleomycin as a monotherapy and in combination with sirolimus exerted inhibitory effects on the proliferation of CD31^+^ NICHECs. However, escalating the drug concentration did not significantly augment this inhibitory effect. These findings offer novel insights into the limited efficacy often observed with sclerosing agents and pharmacotherapy in clinical practice for CH. Nonetheless, further investigation is required to elucidate the underlying mechanisms. Our xenograft model predominantly features proliferative CD31^+^ NICHECs, which may not accurately reflect the quiescent state of human NICH. The microenvironment within the nude mouse xenograft, characterized by hypoxia and murine cytokines, may artificially enhance drug sensitivity. In contrast, the mature vascular structures and fibrotic stroma present in human NICH could impede drug penetration. Future research should focus on evaluating drug efficacy using patient-derived xenografts that preserve human stromal components. Although this study describes the preliminary effects of drugs, detailed molecular mechanism or mechanistic studies are lacking, which constrains the understanding of how these drugs work. Despite these shortcomings, our study provides a new strategy for mechanistic research and drug validation for NICH.

## Data Availability

The original contributions presented in the study are included in the article/supplementary material, further inquiries can be directed to the corresponding authors.
